# Dietary Polyamine Intake Across Age Groups in Spain: A Comprehensive Assessment

**DOI:** 10.3390/nu18101584

**Published:** 2026-05-16

**Authors:** Natalia Toro-Funes, Oriol Comas-Basté, Mariluz Latorre-Moratalla, Maria Teresa Veciana-Nogués, M. Carmen Vidal-Carou

**Affiliations:** 1 Bioactive Amines and Polyamines in Food Research Group (BAPfood), Department of Nutrition, Food Sciences and Gastronomy, Food and Nutrition Torribera Campus, University of Barcelona, Av. Prat de la Riba 171, 08921 Santa Coloma de Gramenet, Spain; natalia.toro@ub.edu (N.T.-F.); oriolcomas@ub.edu (O.C.-B.); veciana@ub.edu (M.T.V.-N.); mcvidal@ub.edu (M.C.V.-C.); 2 Research Institute of Nutrition and Food Safety (INSA·UB), University of Barcelona, Av. Prat de la Riba 171, 08921 Santa Coloma de Gramenet, Spain

**Keywords:** polyamine, spermidine, spermine, putrescine, elderly, nutrition, healthy aging, Mediterranean diet

## Abstract

Background: Polyamines, including putrescine (PU), spermidine (SPD), and spermine (SPM), are ubiquitous bioactive compounds essential for cell proliferation, genomic stability, autophagy, and the regulation of oxidative and inflammatory responses. Growing evidence, particularly for SPD, suggests that polyamine-rich diets may protect against age-related conditions such as cardiovascular disease, metabolic syndrome, and neurodegenerative disorders. As endogenous polyamine synthesis declines with age, dietary intake becomes increasingly important, especially in older adults. Methods: This study estimated each polyamine (PU, SPD and SPM) and total polyamine intake in the Spanish population using food consumption data from the Spanish Ministry of Agriculture, Fisheries and Food. Intakes were evaluated across four age groups, and major dietary sources were identified. Results: Total polyamine intake increased with age, reaching 393 µmol/day in adults over 65 years. PU accounted for 49% of total intake, followed by SPD (29%) and SPM (22%). Plant-based foods were the primary contributors to SPD intake, particularly vegetables (36%), fruits (26%), and cereals (18%). PU intake was also predominantly plant-derived, mainly from fruits (58%) and vegetables (23%), whereas SPM intake was largely associated with meat products (59%). A theoretical Mediterranean diet model yielded a slightly higher total polyamine intake of 406.6 µmol/day and a substantially greater SPD intake than that observed in older adults (193.99 µmol/day versus 121.62 µmol/day). Conclusions: Overall, estimated polyamine intake in the Spanish population fell below the optimal level of 540 µmol/day proposed in the literature. These findings highlight the need for public health strategies promoting consumption of polyamine-rich foods, particularly vegetables, legumes, whole grains, and fruits, to support healthy aging and reduce the risk of age-related diseases.

## 1. Introduction

Polyamines, including putrescine (PU; butane-1,4-diamine), spermidine (SPD; *N*-(3-aminopropyl)-butane-1,4-diamine), and spermine (SPM; *N*,*N*-bis(3-aminopropyl)-butane-1,4-diamine), are ubiquitous aliphatic compounds essential for all living organisms. In humans, they play key roles in fundamental biological processes such as cell proliferation and differentiation, genomic stability, autophagy, and the regulation of oxidative and inflammatory responses [1,2,3]. Through these functions, polyamines, especially SPD, have been associated with healthy aging and a reduced risk of age-related pathologies, such as cardiovascular diseases, metabolic syndrome, neurodegenerative disorders, and muscle decline [4,5]. Their anti-inflammatory and autophagy-enhancing properties may also support recovery from surgery or intensive treatments such as chemotherapy by promoting cellular repair and immune function [6,7].

The human body maintains its polyamine pool through endogenous synthesis, production by the gut microbiota, and dietary intake [2,8]. However, because endogenous synthesis and tissue concentrations decline with age [7,9], dietary sources become increasingly important, particularly for older adults. In this context, prospective population-based studies of Mediterranean dietary patterns have shown that long-term consumption of polyamine-rich foods is associated with higher circulating polyamine levels and a reduced risk of cardiovascular and other age-related diseases [10,11]. Similarly, higher dietary intake of SPD has been linked to lower cardiovascular and all-cause mortality, as well as reduced cognitive decline and neurodegenerative disorders in individuals over 60 years of age [12,13,14,15].

Despite growing evidence of health benefits, no consensus exists on recommended daily intakes of polyamines or SPD. Ali et al. (2011) proposed a total polyamine intake of 540 µmol/day based on a diet rich in fruits, vegetables, and cereals [16]; however, the few available estimates from different populations suggest that real-life intake is substantially lower [16,17,18,19,20,21].

This study aimed to estimate the daily intake of each polyamine (PU, SPD and SPM) and total polyamines in the Spanish population across different age groups, with a particular focus on older adults, and to identify key dietary sources to inform future dietary recommendations. This study was conducted using a deterministic dietary assessment approach, which represents a well-established methodology in nutritional epidemiology and public health research [22]. In the case of polyamines, few studies have also used this deterministic approach to estimate this dietary intake [11,12,13,17,21].

## 2. Materials and Methods

### 2.1. Polyamine Content and Consumption Data

Polyamine intake was estimated using polyamine content data from a database developed by our research group, which was progressively compiled from analytical data obtained from more than 200 representative foods available on the Spanish market [2,23]. Currently, this database includes 205 types of foods and beverages, with more than 2300 food samples. Polyamine concentrations were determined using consistent analytical methodologies [24]. Polyamine contents expressed as mg/kg were converted to μmol/day for intake estimation.

Food consumption data were obtained from the 2022 Household Food Consumption Panel of the Spanish Ministry of Agriculture, Fisheries and Food (MAPA) [25]. This MAPA database includes 137 representative foods and beverages from the Spanish market, classified into 15 categories, and provides nationally representative consumption data based on a sample of 12,500 households. The dataset covers diverse geographic regions, cities and towns of variable sizes, and a range of socioeconomic groups.

To link food consumption with polyamine content, food items from the MAPA database were matched with the corresponding mean polyamine contents of our database. Fresh and frozen foods were considered within the same category, as freezing does not affect polyamine content [23,26]. Overall, a total of 2188 samples, corresponding to the 15 food MAPA categories, were included in the assessment of polyamine intake.

### 2.2. Assessment of Dietary Polyamine Intake in the Spanish Population

Polyamine intake (μmol/day) was estimated using a deterministic approach by multiplying the mean polyamine content (μg/g) of each food or beverage by its corresponding mean consumption (g/day). Estimates were calculated for four age groups: 18–35, 35–49, 50–64, and >65 years. Total polyamine intake was obtained by summing the contributions of all food and beverage categories.

Additionally, the mean daily intake of each polyamine (SPD, SPM and PU) and total polyamines was estimated using a theoretical Mediterranean diet model proposed by Davis et al. (2015) [27], based on dietary data (with 12 food groups) collected from 15 populations over a 46-year period (1960–2006).

### 2.3. Statistical Analysis

After verifying the assumptions of normality and homogeneity of variances (using Shapiro–Wilk and Levene’s tests, respectively), one-way analysis of variance (ANOVA), followed by Tukey’s post hoc test when appropriate, was used to assess differences in the relative contribution of each polyamine (SPD, SPM and PU) to total polyamine intake among age groups, as this approach allows comparison of mean values across more than two independent groups. A *p*-value of less than 0.05 was considered statistically significant. All statistical analyses were performed using IBM SPSS Statistics 27.0 (IBM Corporation, Armonk, NY, USA). Moreover, descriptive comparisons were also made between the estimated intake and that derived from the Mediterranean diet model, used as a reference dietary pattern.

## 3. Results and Discussion

### 3.1. Polyamine Content in Food Categories

Table 1 presents polyamine concentrations (mg/kg) in the 15 MAPA food categories. Among the three polyamines analyzed, SPD has received particular attention due to its role in cellular homeostasis, autophagy induction, and potential anti-aging effects [2,3,4,5,6,7,8,9,10,11,28,29,30].

Legumes, nuts, and vegetables were identified as the food categories with the highest SPD content, with SPD generally predominating in plant-derived foods. Substantial variability in SPD levels was observed not only across food categories but also within the same category, and even among samples of the same food item. The relatively high standard deviations observed in some food categories reflect the intrinsic variability of polyamine content within heterogeneous food groups, rather than analytical uncertainty. This variability arises from origin, cultivation conditions, harvesting, and storage practices [2]. Within legumes, green peas (55 ± 20 µg/g; mean ± SD) and lentils (44 ± 42 µg/g) exhibited the highest SPD levels, while mushrooms (129 ± 15 µg/g) were the richest source among vegetables. These findings are consistent with previous reports identifying mushrooms and legumes as major dietary sources of SPD [2]. From a nutritional perspective, these results are particularly relevant given the growing evidence linking dietary SPD to improved cardiovascular function and increased lifespan [11,12,13,29,30].

In contrast, SPM was generally more abundant than SPD in animal-derived foods (Table 1). Fish and seafood contained lower SPM levels than meat, but higher SPD concentrations. Dairy products and eggs showed lower SPM levels than meat and fish, while SPD contents were similar. Overall, animal-derived foods appear to contribute more significantly to SPM intake than to SPD intake, although notable variability exists across categories. Among plant-based foods, legumes and nuts also provided substantial amounts of SPM, albeit with considerable variability.

PU was widely distributed across both plant- and animal-based foods, with marked within-category variability. Legumes, vegetables, and fruits emerged as the main contributors to PU intake. PU occurs naturally in foods as part of their physiological composition; however, its concentration can increase significantly in fermented products due to the activity of aminogenic microorganisms [26]. This dual origin, physiological and fermentation-related, may help explain the wide variability observed in PU concentrations.

The heterogeneous distribution of polyamines across food groups highlights the importance of considering both animal and plant sources when evaluating dietary intake and potential health implications. From a physiological perspective, polyamine metabolism in the human body begins with PU, which is synthesized endogenously via ornithine decarboxylation and subsequently converted into SPD and SPM. As endogenous synthesis declines with age, adequate dietary intake becomes increasingly important, particularly in older populations.

### 3.2. Total Polyamine Intake in the Spanish Population

Table 2 presents the mean estimated daily intake (µmol/day) of PU, SPD, SPM, and total polyamines in different age groups as well as the estimated contribution of each food category to overall polyamine intake. Detailed information on the foods included in each food category and the corresponding polyamine intake estimates for individual food items is provided in Supplementary Table S1.

Total polyamine intake increased with age, ranging from 127.1 µmol/day in individuals under 35 years to 393.0 µmol/day in adults over 65 years. This age-related increase may be partly explained by greater adherence to traditional Mediterranean dietary patterns among older adults, whereas younger individuals tend to follow less balanced diets with lower consumption of plant-based foods [31,32], which are the primary sources of polyamines.

In all age groups, fruits and vegetables were the main dietary sources of polyamines, with the highest consumption observed in the elderly. Cereals, meat products, and dairy also contributed significantly, whereas fish, seafood, and eggs had a minor impact. Within the dairy category, cheese was the main contributor, clearly more than other dairy products (mean intake: 1.3 µmol/day; SD = 6.3 µmol/day). In particular, ripened cheeses accounted for approximately 4–10% of total daily polyamine intake, depending on the age group (Table S1).

The total polyamine intake estimated for Spanish adults over 65 years (393 µmol/day) is comparable to that reported by Cantabrana et al. [21] for a Spanish cohort aged over 60 years (430 µmol/day). However, that study was based on a relatively small, non-random sample (15 individuals > 70 years and 61 aged 60–69 years) from a single region. The present analysis, based on a nationally representative dataset (*n* = 12,500 households), supports and extends these earlier findings despite methodological differences.

Reported polyamine intake in adult populations (15–80 years) varies across countries: approximately 200 µmol/day in Japan [17], 250 µmol/day in the United States [18], and 315–388 µmol/day across several European countries [19], while a lower value of 140 µmol/day has been reported for Turkey [20]. The intake observed in older Spanish adults in the present study is comparable to several of these values.

Nevertheless, comparisons between studies require caution due to differences in dietary patterns, variability in polyamine content of foods, and methodological heterogeneity. Studies differ in age ranges (e.g., 15–69 years in Japan [17], 40–80 years in the United States [18], and 20–60 years in Turkey [20]), dietary assessment methods (national surveys, food frequency questionnaires, 7-day food records, and 24-h dietary recalls), and sources of polyamine composition data. Some studies generated their own polyamine content data [17,18], whereas others relied on published datasets [20].

To our knowledge, all previous polyamine intake estimates (including the present one) are based on raw food composition, without accounting for potential losses during cooking. However, culinary processing is known to affect polyamine levels: boiling and grilling can reduce content by up to 64%, whereas microwave and sous-vide methods appear to have minimal impact [23,33,34]. Therefore, dietary polyamine exposure may be overestimated for certain foods, depending on cooking practices. Future nutritional studies should account for the effects of food processing and develop standardized methods to improve the accuracy of intake estimation.

Currently, no scientific consensus exists regarding recommended daily polyamine intake, despite accumulating evidence supporting their role, especially that of SPD, in modulating oxidative stress, inflammation, and age-related diseases [4,5,29,30]. Ali et al. (2011) proposed an intake of approximately 540 µmol/day based on a healthy dietary pattern rich in fruits, vegetables, and cereals [16]. In this context, the estimated intake in the Spanish population falls below the proposed level in all age groups. Promoting the consumption of polyamine-rich food may therefore represent a key public health strategy, particularly for older adults, pending confirmation from outcome-based studies. Enhanced dietary intake may help compensate for age-related declines in endogenous polyamine synthesis and contribute to improved cellular function, reduced inflammation, and healthier aging [3,4,5]. Nowadays, commercially available SPD supplements may represent an additional source of SPD. In older people, few randomized controlled trials suggest that daily supplementation of less than 5 mg of food-derived SPD extracts may improve cognitive function [15,35,36,37,38]. Higher doses of purified SPD (15–40 mg/day) have also demonstrated short-term safety [39,40]; however, further studies are required to establish long-term efficacy and safety before population-level recommendations can be made.

Previous studies have generally not distinguished between each polyamine, despite their distinct physiological roles. Given the growing evidence supporting the specific role of SPD in healthy aging, its targeted assessment is particularly relevant, as it enables the identification of key dietary sources and supports the development of more precise nutritional recommendations.

### 3.3. Each Dietary Polyamine Intake in the Spanish Population

One-way ANOVA showed no significant differences (F = 1.21; *p* > 0.05) among age groups in the relative contribution of each polyamine to total polyamine intake. Consequently, no post hoc analyses were performed. Overall, the Spanish population, PU, SPD, and SPM accounted for 49%, 29%, and 22% of total polyamine intake, respectively, across all groups.

**Figure 1:**
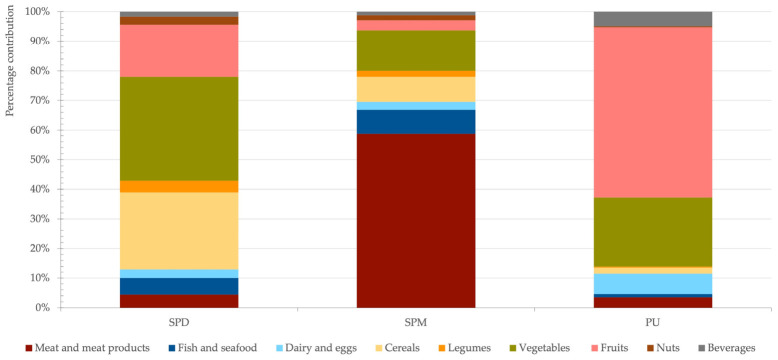
Percentage contribution of each food category to the total daily intake of spermidine (SPD), spermine (SPM) and putrescine (PU).

As shown in Figure 1, cereals, vegetables, and fruits were the main contributors to SPD intake in all age groups, accounting for approximately 30%, 35%, and 20% of total SPD intake, respectively. Together, these three food groups provided more than 75% of total dietary SPD (Figure 1). Although some legumes, such as green peas and lentils, contain high SPD concentrations, their overall contribution was limited due to low consumption levels. This finding highlights that the dietary impact of a given food depends not only on its nutrient content but also on consumption patterns shaped by cultural, economic, and behavioral factors.

Meat and meat products were the primary contributors to SPM intake, accounting for 59% of the total (Figure 1), with chicken, pork, and beef as the main sources. In contrast, PU intake was predominantly derived from plant-based foods, particularly fruits and vegetables, which contributed 58% and 23%, respectively (Figure 1). Among fruits, oranges were the leading contributors, accounting for up to 21% of total daily PU intake in older adults. These results are consistent with previous studies identifying fruits and vegetables as the main dietary sources of PU [18,26,28].

### 3.4. Assessment of Total Dietary Polyamines in a Mediterranean Diet

In the absence of established dietary recommendations for polyamines, the Mediterranean diet as used as a reference model to approximate a desirable intake. Adherence to this dietary pattern is consistently associated with reduced cardiovascular risk and increased longevity, benefits partly attributed to its high content of bioactive compounds, including polyamines [10]. Accordingly, it provides a suitable benchmark against which to evaluate the observed polyamine intake levels in the Spanish population.

The Mediterranean diet model developed by Davis et al. (2015) [27] was selected for this purpose. Based on dietary data from 15 populations collected over a 46-year period (1960–2006), this model offers a comprehensive and historically grounded characterization of the Mediterranean dietary pattern. It includes detailed daily consumption values for 12 food groups: bread (298.6 g/day), cereals excluding bread (305.8 g/day), legumes (35.6 g/day), potatoes (125.8 g/day), all vegetables (374.9 g/day), fruits and nuts (268.7 g/day), meat and meat products (105.1 g/day), cheese (21.9 g/day), dairy excluding cheese (213.6 g/day), eggs (23 g/day), olive oil (44 g/day), and fish products (50 g/day).

Polyamine intake (μmol/day) was estimated by multiplying the mean content of each polyamine (μg/g) in each food group by the corresponding recommended daily consumption (g/day). To enable direct comparison, polyamine intake in the Spanish elderly population, who showed the highest intake among all age groups, was recalculated using the same 12 food categories defined by Davis et al. [27].

The estimated daily intakes for both the Mediterranean diet and the Spanish elderly population are presented in Table 3, allowing assessment of whether observed intake levels align with those expected from a nutritionally optimal dietary pattern.

The Mediterranean diet model yielded an estimated total polyamine intake of 406.6 µmol/day. In contrast, the mean intake observed among older adults in this study was 300.8 µmol/day, which is substantially lower. This value is also below the 393 µmol/day estimated using the 15 food categories of the MAPA database. Likewise, the Mediterranean dietary model implies a higher intake of polyamines compared to the reported intakes in other countries, such as Japan, the United States, or Turkey [17,18,20].

In addition to total polyamines, the Mediterranean diet model provided a markedly higher SPD intake (193.99 µmol/day) compared with that of Spanish adults over 65 years (121.62 µmol/day). This difference highlights the value of the Mediterranean dietary pattern as a rich source of SPD. As suggested by Binh et al. [10], the well-established health benefits of this diet may be partially explained by its high polyamine content.

Given that SPD is one of the predominant polyamines found in plant-derived foods, increased consumption of these foods naturally leads to higher SPD intake [2,10]. This is especially relevant for older adults, as endogenous SPD synthesis declines with age. Therefore, promoting a diet rich in plant-based foods may help compensate for this age-related reduction.

Among commonly consumed food groups, several items stand out as important sources of polyamines: oranges, mandarins, and bananas (fruits); mushrooms (vegetables); lentils and green peas (legumes); and whole grains (cereal products). Increasing the consumption of these foods may enhance both total polyamine and particularly SPD intake, thereby improving nutritional support during aging. Therefore, according to our results, ensuring an adequate SPD intake is a key factor in following a dietary pattern characterized by high plant-based food consumption, such as the Mediterranean diet.

### 3.5. Limitations

The MAPA database is based on household food consumption data and does not account for meals consumed outside the home, such as in restaurants or catering establishments. This limitation may differentially affect population subgroups, as younger individuals, particularly those under 35 years of age, tend to consume a smaller proportion of meals at home compared with older adults. Consequently, polyamine intake may be underestimated to a greater extent in younger age groups.

Additionally, the intake estimations did not account for the impact of cooking and food processing methods on polyamine content. Dietary assessments are typically based on raw food composition data; however, culinary practices may alter polyamine levels, potentially resulting in lower actual exposure than estimated. Nevertheless, this limitation is consistent with the inherent uncertainty commonly associated with dietary intake evaluations.

## 4. Conclusions

This study provides a detailed assessment of dietary polyamine intake in the Spanish population, with a particular focus on SPD. The findings confirm that polyamines are widely distributed across commonly consumed foods, with vegetables, cereals, and fruits representing the main contributors to SPD intake.

Older adults (>65 years) exhibited the highest total polyamine intake, averaging 393 µmol/day, with SPD contributing 92.5 µmol/day, which corresponds to 29% of total polyamine intake. However, SPD intake in this group was lower than that estimated using a Mediterranean diet model, suggesting potential for dietary improvement. Given that endogenous polyamine synthesis declines with age, adequate dietary intake of SPD could become increasingly important for maintaining cellular health, modulating inflammation, and reducing the risk of age-related diseases, including cardiovascular and neurodegenerative disorders. Therefore, further confirmation of the evidence regarding the role of polyamines in healthy aging is warranted. If the findings from the currently available studies are substantiated, the potential role of dietary strategies aimed at increasing polyamine intake, preferably through polyamine-rich foods rather than supplementation, could be explored as a possible public health approach.

## Figures and Tables

**Table 1:** Polyamine concentrations (mean ± SD) in MAPA food categories.

	Polyamine Concentrations (mg/kg or µg/g)		
Food Product	Putrescine	Spermidine	Spermine
Fresh meat (*n* = 225)	3.25 ± 5.40	3.97 ± 2.47	36.18 ± 15.55
Processed meat (*n* = 487)	36.67 ± 40.92	4.17 ± 1.61	22.48 ± 9.12
Fish (*n* = 223)	2.28 ± 2.70	5.62 ± 2.08	8.63 ± 4.62
Seafood (*n* = 39)	7.89 ± 10.01	11.99 ± 14.96	6.64 ± 6.79
Dairy (*n* = 125)	25.46 ± 32.70	4.87 ± 5.41	1.51 ± 1.67
Eggs (*n* = 16)	nd	3.61 ± 2.45	4.48 ± 3.47
Cereals (*n* = 65)	1.80 ± 1.21	10.24 ± 7.94	3.59 ± 2.90
Legumes (*n* = 34)	23.83 ± 39.18	64.49 ± 63.93	24.25 ± 22.66
Oil (*n* = 12)	2.64 ± 1.33	4.2 ± 2.41	5.73 ± 4.04
Vegetables (*n* = 327)	12.82 ± 19.27	17.14 ± 25.75	4.16 ± 5.04
Fruits (*n* = 142)	13.51 ± 27.67	5.42 ± 3.69	1.02 ± 0.94
Nuts (*n* = 71)	5.10 ± 4.69	25.10 ± 6.79	12.03 ± 6.08
Beverages (*n* = 366)	5.61 ± 11.60	2.19 ± 2.42	2.11 ± 5.00
Dressings (*n* = 11)	0.97 ± 0.64	7.99 ± 14.71	0.82 ± 0.86
Chocolates (*n* = 45)	0.47 ± 0.43	2.49 ± 1.94	1.95 ± 1.50

nd: not detected. *n*: number of food items analyzed per food group.

**Table 2:** Estimated daily polyamine intake (µmol/day) by food category and age group in the Spanish population.

	Estimated Daily Polyamine Intake (µmol/day) *															
	Population Aged <35 Years				Population Aged 35–49 Years				Population Aged 50–64 Years				Population Aged >65 Years			
Food Product	PU	SPD	SPM	Sum	PU	SPD	SPM	Sum	PU	SPD	SPM	Sum	PU	SPD	SPM	Sum
Fresh meat	0.56	1.57	11.12	13.24	0.64	1.74	12.67	15.06	1.01	2.65	19.51	23.17	1.31	3.39	24.57	29.27
Processed meat	4.03	0.48	2.34	6.85	5.61	0.59	2.85	9.05	7.34	0.65	3.23	11.23	7.59	0.66	3.25	11.50
Fish	0.47	0.59	0.70	1.76	0.69	0.83	0.93	2.45	1.30	1.49	1.38	4.17	1.93	2.45	2.51	6.89
Seafood	0.14	0.44	0.23	0.81	0.21	0.74	0.37	1.32	0.43	1.61	0.79	2.83	0.72	2.72	1.31	4.74
Dairy	11.84	1.11	0.27	13.21	12.13	1.22	0.28	13.63	15.39	1.47	0.35	17.21	17.22	1.82	0.42	19.46
Eggs	0.00	0.40	0.36	0.76	0.00	0.41	0.36	0.77	0.00	0.63	0.56	1.19	0.00	0.97	0.84	1.81
Cereals	2.04	7.44	1.66	11.14	2.64	10.51	2.01	15.16	4.13	17.02	3.03	24.17	5.55	23.76	4.00	33.32
Legumes	0.38	1.61	0.44	2.43	0.35	1.57	0.42	2.35	0.54	2.47	0.68	3.69	0.79	3.54	0.97	5.31
Oil	0.22	0.19	0.19	0.60	0.34	0.18	0.18	0.71	0.70	0.25	0.25	1.20	1.18	0.36	0.35	1.89
Vegetables	20.30	11.74	2.12	34.16	23.46	13.13	2.48	39.07	38.52	21.28	4.06	63.87	58.57	32.39	6.46	97.42
Fruits	30.70	4.24	0.45	35.40	41.64	5.52	0.56	47.71	78.19	9.55	0.94	88.68	144.62	16.12	1.59	162.34
Nuts	0.32	1.03	0.32	1.67	0.41	1.33	0.42	2.16	0.59	1.96	0.62	3.17	0.78	2.50	0.80	4.08
Beverages	3.55	0.85	0.32	4.72	4.91	0.98	0.37	6.27	9.25	1.31	0.54	11.10	12.39	1.56	0.57	14.53
Dressings	0.04	0.12	0.02	0.19	0.04	0.11	0.02	0.17	0.05	0.11	0.02	0.18	0.07	0.12	0.02	0.21
Chocolates	0.03	0.09	0.05	0.17	0.04	0.11	0.06	0.21	0.04	0.13	0.08	0.25	0.04	0.15	0.08	0.27
Total Polyamine Intake	74.61	31.90	20.58	127.09	93.11	38.98	23.98	156.07	157.48	62.59	36.04	256.11	252.77	92.51	47.74	393.03

PU = putrescine; SPD = spermidine; SPM = Spermine. * Values correspond to mean estimated intakes derived from deterministic calculation using mean food consumption and mean polyamine content data.

**Table 3:** Estimated daily polyamine intake (µmol/day) for a Mediterranean diet and for the Spanish elderly population.

	Estimated Intake (µmol/day) for a Mediterranean Diet				Estimated Intake (µmol/day) for the Spanish Elderly			
	Putrescine	Spermidine	Spermine	Total	Putrescine	Spermidine	Spermine	Total
Bread	5.25	21.83	1.70	28.78	5.71	23.76	1.85	31.32
Other cereals	15.67	73.71	16.81	106.19	3.74	17.60	4.01	25.35
Legumes	1.74	8.34	2.29	12.36	0.70	3.34	0.92	4.96
Potato	5.91	7.78	1.61	15.30	5.17	6.80	1.41	13.38
Vegetables	57.73	52.80	7.94	118.47	46.66	42.67	6.42	95.75
Fruits and nuts	33.35	19.56	4.96	57.87	21.15	12.40	3.15	36.71
Meat and meat products	23.80	3.01	15.23	42.04	31.62	3.99	20.24	55.86
Cheese	10.02	1.04	0.16	11.21	12.01	1.24	0.19	13.44
Other dairy products	1.70	2.65	1.71	6.06	2.71	4.24	2.73	9.69
Eggs	0.00	0.58	0.51	1.09	0.00	0.97	0.84	1.81
Olive oil	1.32	0.00	0.00	1.32	1.67	0.00	0.00	1.67
Fish products	1.37	2.52	2.05	5.94	2.50	4.59	3.75	10.84
Total of all food categories	157.85	193.81	54.97	406.62	133.64	121.62	45.51	300.77

## Data Availability

The original contributions presented in this study are included in the article/Supplementary Material. Further inquiries can be directed to the corresponding author.

## Supplementary Materials

The following supporting information can be downloaded at: https://www.mdpi.com/article/10.3390/nu18101584/s1, Table S1: Estimation of each and total polyamine daily intake (µmol/day) per food in the Spanish population.

## Abbreviations

The following abbreviations are used in this manuscript:

SPM	Spermine
SPD	Spermidine
PU	Putrescine
MAPA	Spanish Ministry of Agriculture, Fisheries and Food
