# Supplementary material for: Dietary Polyamine Intake Across Age Groups in Spain: A Comprehensive Assessment
Source: Nutrients. 2026 May 16;18(10):1584. doi: 10.3390/nu18101584 (PMC13209393; doi:10.3390/nu18101584)
Supplement: Supplementary file 1 [file nutrients-18-01584-s001.zip › nutrients-4277430-supplementary.pdf]

Table S1. Estimation of each and total polyamine daily intake (µmol/day) per food in the Spanish population.

	Estimated daily intake (µmol/día)															
	Population aged < 35 years				Population aged 35 - 49 years				Population aged 50 - 64 years				Population aged > 65 years			
**FOOD PRODUCTS**	**PU**	**SPD**	**SPM**	**Sum**	**PU**	**SPD**	**SPM**	**Sum**	**PU**	**SPD**	**SPM**	**Sum**	**PU**	**SPD**	**SPM**	**Sum**
Meat products																
Fresh meat																
Sausages	0.06	0.02	0.05	0.13	0.10	0.03	0.07	0.20	0.15	0.04	0.12	0.31	0.19	0.05	0.15	0.39
Beef	0.15	0.11	1.28	1.54	0.17	0.13	1.47	1.77	0.29	0.22	2.50	3.00	0.40	0.31	3.50	4.22
Chicken	0.28	1.22	6.96	8.46	0.30	1.31	7.50	9.11	0.44	1.93	11.03	13.40	0.53	2.38	13.31	16.22
Lamb	0.01	0.04	0.26	0.31	0.01	0.04	0.30	0.36	0.03	0.10	0.64	0.77	0.06	0.19	1.28	1.54
Pork	0.02	0.16	2.38	2.55	0.02	0.20	3.12	3.34	0.03	0.32	4.93	5.29	0.04	0.40	5.95	6.38
Turkey	0.04	0.03	0.19	0.25	0.04	0.03	0.21	0.28	0.06	0.04	0.30	0.40	0.08	0.05	0.38	0.51
Meat products																
Cured ham	0.19	0.17	0.77	1.14	0.21	0.18	0.83	1.23	0.19	0.17	0.77	1.14	0.17	0.16	0.70	1.03
Spanish chorizo	2.57	0.07	0.26	2.90	3.38	0.10	0.35	3.83	4.71	0.13	0.48	5.33	4.94	0.14	0.51	5.59
Salchichon	0.53	0.02	0.06	0.61	0.85	0.04	0.10	0.99	1.08	0.05	0.12	1.26	1.08	0.05	0.12	1.25
Fuet/Longaniza	0.62	0.04	0.12	0.78	1.01	0.06	0.20	1.28	1.18	0.07	0.23	1.49	1.22	0.08	0.24	1.54
Sausage, wiener	0.04	0.06	0.19	0.30	0.04	0.07	0.20	0.31	0.03	0.05	0.17	0.26	0.03	0.04	0.13	0.21
Cooked ham	0.09	0.11	0.92	1.12	0.11	0.14	1.17	1.42	0.14	0.17	1.45	1.76	0.15	0.19	1.55	1.88
Total Meat Products	4.59	2.05	13.45	20.09	6.25	2.33	15.52	24.11	8.35	3.30	22.75	34.39	8.90	4.05	27.82	40.77
Fish and seafood																
Fish																
Hake	0.02	0.04	0.07	0.13	0.04	0.05	0.11	0.20	0.07	0.10	0.20	0.37	0.12	0.18	0.35	0.65
Sardine	0.02	0.05	0.05	0.13	0.03	0.08	0.09	0.21	0.08	0.20	0.21	0.48	0.15	0.37	0.39	0.92
Tuna	0.03	0.15	0.30	0.48	0.03	0.17	0.33	0.53	0.08	0.20	0.21	0.49	0.06	0.32	0.60	0.97

Trout	0.00	0.00	0.01	0.01	0.01	0.01	0.01	0.03	0.01	0.02	0.03	0.05	0.02	0.03	0.04	0.09
Sole	0.00	0.00	0.00	0.01	0.01	0.01	0.01	0.02	0.01	0.02	0.02	0.05	0.02	0.03	0.03	0.08
Cod	0.00	0.04	0.00	0.04	0.00	0.07	0.00	0.07	0.00	0.16	0.00	0.16	0.00	0.27	0.00	0.27
Mackerel	0.14	0.03	0.01	0.18	0.23	0.06	0.02	0.31	0.41	0.10	0.03	0.55	0.60	0.15	0.05	0.80
Sea bream	0.03	0.07	0.06	0.17	0.04	0.09	0.08	0.22	0.07	0.15	0.14	0.35	0.09	0.19	0.17	0.45
Salmon	0.11	0.07	0.06	0.24	0.15	0.09	0.07	0.31	0.26	0.15	0.13	0.54	0.34	0.20	0.17	0.72
Other oily fish	0.03	0.03	0.04	0.11	0.04	0.04	0.05	0.14	0.08	0.09	0.10	0.27	0.12	0.13	0.16	0.40
Other white fish	0.00	0.01	0.01	0.03	0.01	0.02	0.02	0.04	0.02	0.05	0.04	0.10	0.03	0.08	0.07	0.18
Other fish	0.07	0.08	0.08	0.23	0.11	0.13	0.14	0.38	0.22	0.26	0.27	0.75	0.39	0.48	0.48	1.35
Seafood																
Scallops	0.00	0.01	0.03	0.05	0.00	0.02	0.04	0.07	0.01	0.04	0.10	0.15	0.02	0.08	0.16	0.26
Muscles	0.02	0.22	0.07	0.30	0.03	0.38	0.13	0.54	0.06	0.84	0.29	1.19	0.10	1.45	0.49	2.04
Squid	0.01	0.03	0.02	0.06	0.02	0.05	0.03	0.09	0.03	0.09	0.06	0.18	0.04	0.14	0.09	0.27
Shrimp	0.09	0.08	0.05	0.22	0.12	0.12	0.07	0.31	0.24	0.23	0.13	0.60	0.41	0.40	0.23	1.04
Other seafoods	0.02	0.10	0.05	0.17	0.04	0.17	0.09	0.31	0.09	0.40	0.22	0.70	0.14	0.65	0.34	1.13
Total Fish and seafood	0.60	1.03	0.93	2.56	0.90	1.57	1.29	3.77	1.73	3.09	2.17	6.99	2.64	5.17	3.82	11.63
Dairy and eggs																
Cow milk	0.13	0.08	0.00	0.21	0.18	0.11	0.00	0.29	0.21	0.12	0.00	0.33	0.29	0.18	0.00	0.47
Fermented milk, yogurs	1.07	0.36	0.07	1.50	1.32	0.44	0.09	1.85	1.46	0.49	0.10	2.04	1.97	0.67	0.13	2.77
Buttermilk	0.00	0.02	0.02	0.04	0.00	0.02	0.02	0.04	0.00	0.03	0.02	0.05	0.00	0.04	0.03	0.07
Fresh cheese	0.02	0.01	0.01	0.04	0.02	0.01	0.01	0.04	0.03	0.01	0.01	0.05	0.04	0.02	0.01	0.06
Hard cheese	8.33	0.45	0.15	8.93	8.53	0.46	0.15	9.14	11.28	0.61	0.20	12.09	12.41	0.69	0.22	13.32
Goat cheese	0.20	0.02	0.00	0.22	0.24	0.02	0.00	0.27	0.41	0.04	0.01	0.45	0.46	0.04	0.01	0.51
Blue cheese	0.06	0.02	0.00	0.08	0.08	0.02	0.00	0.10	0.12	0.03	0.00	0.16	0.16	0.04	0.00	0.20
Other cheeses	2.01	0.15	0.02	2.18	1.76	0.13	0.02	1.91	1.88	0.14	0.02	2.05	1.89	0.15	0.02	2.06
Eggs	0.00	0.40	0.36	0.76	0.00	0.41	0.36	0.77	0.00	0.63	0.56	1.19	0.00	0.97	0.84	1.81
Total Dairy and eggs	11.84	1.51	0.62	13.97	12.13	1.62	0.65	14.40	15.39	2.10	0.91	18.40	17.22	2.79	1.26	21.28
Cereals																

Bread	0.95	4.45	0.68	6.08	1.65	7.73	1.18	10.56	2.89	13.53	2.06	18.47	4.06	19.42	2.89	26.36
Whole grain bread	0.09	0.34	0.01	0.44	0.13	0.48	0.01	0.62	0.26	0.95	0.03	1.24	0.40	1.53	0.04	1.98
Cookies	0.00	0.06	0.01	0.07	0.00	0.08	0.01	0.10	0.00	0.08	0.01	0.10	0.00	0.10	0.02	0.12
Breakfast cereals	0.11	0.77	0.15	1.03	0.10	0.72	0.14	0.97	0.10	0.72	0.14	0.96	0.10	0.70	0.13	0.93
Rice	0.22	0.13	0.17	0.52	0.19	0.11	0.15	0.44	0.24	0.14	0.18	0.56	0.29	0.17	0.23	0.69
Pasta	0.54	1.19	0.44	2.18	0.48	1.05	0.39	1.93	0.54	1.18	0.44	2.16	0.55	1.23	0.45	2.23
Wheat flour	0.13	0.49	0.20	0.82	0.09	0.34	0.13	0.56	0.11	0.42	0.17	0.70	0.15	0.61	0.24	1.00
Total Cereals	2.04	7.44	1.66	11.14	2.64	10.51	2.01	15.16	4.13	17.02	3.03	24.17	5.55	23.76	4.00	33.32
Legumes																
Chickpeas	0.12	0.75	0.20	1.07	0.12	0.78	0.21	1.11	0.20	1.29	0.34	1.83	0.27	1.78	0.46	2.52
Beans	0.02	0.17	0.13	0.32	0.01	0.15	0.11	0.27	0.02	0.25	0.19	0.46	0.03	0.40	0.29	0.72
Lentils	0.19	0.62	0.10	0.91	0.19	0.60	0.09	0.89	0.27	0.86	0.14	1.27	0.37	1.21	0.19	1.77
Other legumes (Soybean)	0.05	0.06	0.02	0.13	0.03	0.04	0.01	0.08	0.05	0.06	0.02	0.13	0.12	0.15	0.04	0.31
Total Legumes	0.38	1.61	0.44	2.43	0.35	1.57	0.42	2.35	0.54	2.47	0.68	3.69	0.79	3.54	0.97	5.31
Oil																
Olive oil	0.22	0.00	0.00	0.22	0.34	0.00	0.00	0.34	0.70	0.00	0.00	0.70	1.18	0.00	0.00	1.18
Oils	0.00	0.19	0.19	0.38	0.00	0.18	0.18	0.36	0.00	0.25	0.25	0.50	0.00	0.36	0.35	0.71
Total oil	0.22	0.19	0.19	0.60	0.34	0.18	0.18	0.71	0.70	0.25	0.25	1.20	1.18	0.36	0.35	1.89
Vegetables																
Potatoes	2.22	2.86	0.60	5.69	2.65	3.42	0.72	6.80	3.89	5.01	1.06	9.96	5.12	6.75	1.40	13.27
Tomato	5.22	0.58	0.01	5.82	6.10	0.68	0.01	6.79	9.14	1.02	0.02	10.18	13.22	1.51	0.03	14.76
Onion	0.00	0.04	0.00	0.04	0.00	0.04	0.00	0.05	0.01	0.07	0.00	0.08	0.01	0.11	0.00	0.13
Garlic	0.10	0.19	0.09	0.38	0.12	0.23	0.10	0.45	0.22	0.42	0.19	0.83	0.41	0.81	0.36	1.57
Cabbage	0.06	0.23	0.03	0.32	0.08	0.32	0.04	0.44	0.16	0.61	0.08	0.85	0.32	1.25	0.15	1.72
Cucumber	0.17	0.15	0.01	0.33	0.20	0.17	0.01	0.38	0.35	0.30	0.02	0.67	0.59	0.51	0.04	1.15
Green beans	0.32	0.20	0.04	0.56	0.44	0.28	0.05	0.76	0.75	0.48	0.09	1.32	1.35	0.87	0.15	2.37
Pepper	6.90	0.45	0.26	7.62	7.95	0.52	0.30	8.77	14.31	0.94	0.54	15.80	22.39	1.50	0.85	24.74
Mushroom	0.05	2.73	0.05	2.82	0.04	2.47	0.04	2.55	0.06	3.93	0.07	4.06	0.08	4.76	0.08	4.92
Lettuce	0.17	0.29	0.00	0.46	0.22	0.37	0.00	0.59	0.38	0.64	0.00	1.02	0.61	1.06	0.00	1.67

Asparragus	0.19	0.22	0.03	0.43	0.22	0.26	0.04	0.52	0.43	0.50	0.07	1.00	0.69	0.82	0.11	1.61
Spinach	0.09	0.33	0.03	0.44	0.10	0.40	0.03	0.53	0.22	0.83	0.07	1.12	0.40	1.55	0.13	2.07
Chard	0.04	0.05	0.00	0.09	0.07	0.07	0.00	0.14	0.17	0.20	0.01	0.38	0.31	0.36	0.02	0.69
Sweet corn	0.63	0.07	0.00	0.70	0.51	0.05	0.00	0.56	0.52	0.06	0.00	0.57	0.50	0.06	0.00	0.56
Eggplant	0.73	0.07	0.00	0.80	0.89	0.08	0.01	0.97	1.66	0.15	0.01	1.82	2.55	0.23	0.02	2.80
Carrot	0.14	0.24	0.04	0.42	0.15	0.25	0.04	0.44	0.23	0.37	0.06	0.66	0.34	0.57	0.09	0.99
Zuchinni	0.47	0.33	0.04	0.83	0.60	0.42	0.04	1.06	0.96	0.68	0.07	1.72	1.23	0.89	0.09	2.21
Broccoli	0.18	0.09	0.17	0.43	0.19	0.09	0.18	0.46	0.27	0.13	0.25	0.66	0.41	0.21	0.38	1.00
Artichoke	0.07	0.17	0.07	0.31	0.09	0.22	0.10	0.41	0.19	0.46	0.20	0.85	0.44	1.10	0.47	2.00
Celery	0.01	0.02	0.00	0.03	0.01	0.02	0.01	0.04	0.02	0.03	0.01	0.06	0.04	0.07	0.02	0.14
Cauliflower	0.05	0.18	0.02	0.25	0.06	0.24	0.03	0.33	0.10	0.37	0.05	0.51	0.14	0.56	0.07	0.77
Leek	0.03	0.15	0.00	0.17	0.03	0.18	0.00	0.22	0.06	0.31	0.00	0.37	0.09	0.51	0.00	0.60
Green peas	0.62	0.59	0.05	1.26	0.61	0.58	0.05	1.24	0.83	0.80	0.06	1.69	1.24	1.22	0.10	2.56
Spices and condiments	0.02	0.02	0.00	0.04	0.01	0.02	0.00	0.03	0.02	0.02	0.01	0.05	0.02	0.03	0.01	0.06
Other vegetables	1.84	1.51	0.58	3.93	2.12	1.74	0.66	4.52	3.59	2.95	1.13	7.66	6.07	5.10	1.91	13.07
Total Vegetables	20.30	11.74	2.12	34.16	23.46	13.13	2.48	39.07	38.52	21.28	4.06	63.87	58.57	32.39	6.46	97.42
Fruits																
Orange	13.54	0.22	0.01	13.76	19.36	0.31	0.01	19.68	40.90	0.66	0.02	41.58	83.45	1.38	0.04	84.86
Mandarin	6.98	0.12	0.00	7.10	9.55	0.16	0.00	9.71	17.47	0.30	0.00	17.77	30.91	0.54	0.00	31.45
Lemon	0.09	0.05	0.01	0.15	0.10	0.05	0.01	0.17	0.18	0.09	0.02	0.29	0.37	0.19	0.05	0.61
Banana	8.07	1.54	0.12	9.73	9.99	1.90	0.15	12.05	14.83	2.83	0.23	17.88	21.39	4.16	0.33	25.88
Apple	0.05	0.12	0.07	0.23	0.06	0.16	0.10	0.31	0.10	0.27	0.16	0.54	0.18	0.47	0.28	0.93
Pear	0.06	0.09	0.06	0.21	0.08	0.11	0.08	0.27	0.15	0.20	0.15	0.50	0.28	0.39	0.28	0.95
Peach	0.07	0.10	0.00	0.17	0.11	0.14	0.00	0.25	0.23	0.31	0.00	0.53	0.46	0.63	0.00	1.09
Strawberry	0.15	0.15	0.01	0.30	0.20	0.19	0.01	0.40	0.29	0.28	0.02	0.58	0.49	0.48	0.03	1.00
Melon	0.07	0.36	0.00	0.42	0.10	0.55	0.00	0.65	0.19	1.06	0.00	1.26	0.36	1.99	0.00	2.34
Watermelon	0.20	0.70	0.00	0.90	0.27	0.94	0.00	1.20	0.50	1.75	0.00	2.25	0.75	2.67	0.00	3.42
Plum	0.00	0.03	0.01	0.04	0.00	0.03	0.01	0.04	0.00	0.06	0.03	0.09	0.00	0.14	0.06	0.21
Cherry	0.04	0.02	0.00	0.05	0.05	0.02	0.00	0.08	0.12	0.05	0.00	0.17	0.23	0.10	0.00	0.33
Grapes	0.08	0.10	0.03	0.21	0.11	0.13	0.05	0.29	0.19	0.23	0.08	0.50	0.38	0.46	0.16	1.00

Kiwi	0.05	0.10	0.01	0.16	0.06	0.14	0.01	0.22	0.13	0.28	0.03	0.43	0.24	0.55	0.05	0.85
Avocado	0.08	0.13	0.06	0.27	0.08	0.12	0.05	0.25	0.12	0.19	0.09	0.40	0.15	0.24	0.11	0.50
Pineapple	0.08	0.04	0.01	0.13	0.10	0.05	0.01	0.16	0.20	0.11	0.01	0.32	0.26	0.14	0.02	0.42
Raspberry	0.00	0.01	0.00	0.02	0.00	0.02	0.00	0.02	0.00	0.02	0.00	0.02	0.00	0.02	0.00	0.03
Olives	0.13	0.00	0.00	0.14	0.15	0.00	0.00	0.16	0.23	0.01	0.01	0.25	0.33	0.01	0.01	0.35
Grapefruit	0.02	0.01	0.00	0.03	0.03	0.01	0.00	0.04	0.07	0.01	0.00	0.08	0.12	0.03	0.00	0.15
Mango	0.02	0.15	0.01	0.18	0.02	0.17	0.01	0.20	0.04	0.29	0.02	0.34	0.06	0.43	0.02	0.51
Other fruits	0.92	0.23	0.03	1.19	1.21	0.31	0.04	1.56	2.26	0.57	0.08	2.91	4.22	1.09	0.14	5.45
Total Fruits	30.70	4.24	0.45	35.40	41.64	5.52	0.56	47.71	78.19	9.55	0.94	88.68	144.62	16.12	1.59	162.34
Nuts																
Almond	0.01	0.04	0.02	0.07	0.01	0.05	0.03	0.10	0.02	0.11	0.07	0.20	0.03	0.17	0.10	0.31
Peanuts	0.01	0.13	0.02	0.16	0.01	0.16	0.03	0.19	0.02	0.26	0.05	0.33	0.02	0.29	0.05	0.36
Walnut	0.04	0.07	0.03	0.14	0.05	0.08	0.03	0.17	0.11	0.20	0.08	0.38	0.21	0.39	0.15	0.75
Hazelnuts	0.00	0.01	0.00	0.01	0.00	0.02	0.00	0.02	0.00	0.03	0.01	0.04	0.01	0.06	0.01	0.08
Pistachios	0.06	0.07	0.04	0.17	0.07	0.08	0.05	0.21	0.08	0.09	0.06	0.23	0.11	0.13	0.07	0.31
Chestnuts	0.00	0.00	0.00	0.00	0.00	0.00	0.00	0.00	0.00	0.00	0.00	0.00	0.00	0.00	0.00	0.01
Other nuts	0.20	0.71	0.21	1.11	0.26	0.94	0.27	1.47	0.35	1.26	0.37	1.98	0.39	1.45	0.42	2.26
Total Nuts	0.32	1.03	0.32	1.67	0.41	1.33	0.42	2.16	0.59	1.96	0.62	3.17	0.78	2.50	0.80	4.08
Beverages																
Cava	0.03	0.00	0.00	0.03	0.04	0.00	0.00	0.04	0.10	0.00	0.00	0.10	0.16	0.00	0.00	0.16
Red wine	0.57	0.00	0.00	0.57	1.42	0.01	0.00	1.43	3.71	0.02	0.00	3.74	6.37	0.04	0.00	6.41
White wine	0.13	0.01	0.00	0.14	0.15	0.01	0.00	0.16	0.28	0.02	0.00	0.29	0.49	0.03	0.00	0.52
Rosée wine	0.03	0.00	0.00	0.03	0.05	0.00	0.00	0.06	0.22	0.00	0.00	0.22	0.34	0.00	0.00	0.35
Cider	0.01	0.00	0.00	0.01	0.01	0.00	0.00	0.01	0.01	0.00	0.00	0.01	0.01	0.00	0.00	0.01
Beer	2.04	0.14	0.15	2.33	2.52	0.18	0.18	2.88	4.16	0.29	0.30	4.76	3.98	0.28	0.29	4.55
Coffee	0.03	0.04	0.00	0.07	0.04	0.05	0.00	0.09	0.06	0.08	0.00	0.14	0.08	0.10	0.00	0.19
Tea	0.00	0.00	0.01	0.01	0.00	0.00	0.01	0.01	0.00	0.00	0.01	0.02	0.00	0.01	0.01	0.02
Vegetable beverages	0.19	0.56	0.14	0.89	0.22	0.65	0.16	1.03	0.28	0.81	0.21	1.30	0.33	0.99	0.25	1.57
Orange juice	0.18	0.01	0.01	0.20	0.15	0.01	0.01	0.16	0.16	0.01	0.01	0.18	0.29	0.02	0.01	0.32
Peach juice	0.02	0.03	0.01	0.06	0.02	0.03	0.01	0.05	0.01	0.02	0.00	0.04	0.01	0.02	0.00	0.04
Pineapple juice	0.01	0.01	0.00	0.02	0.01	0.01	0.00	0.02	0.01	0.01	0.00	0.02	0.01	0.01	0.00	0.02

Other juices	0.31	0.04	0.01	0.37	0.28	0.04	0.01	0.34	0.24	0.03	0.01	0.28	0.31	0.04	0.01	0.37
Total Beverages	3.55	0.85	0.32	4.72	4.91	0.98	0.37	6.27	9.25	1.31	0.54	11.10	12.39	1.56	0.57	14.53
Dressings																
Ketchup	0.02	0.04	0.01	0.07	0.02	0.04	0.01	0.07	0.01	0.03	0.01	0.05	0.01	0.02	0.01	0.04
Mayonnaise	0.00	0.01	0.00	0.02	0.00	0.01	0.00	0.02	0.00	0.02	0.00	0.02	0.00	0.02	0.00	0.03
Mustard	0.01	0.06	0.00	0.07	0.00	0.05	0.00	0.05	0.00	0.05	0.00	0.06	0.00	0.04	0.00	0.05
Jam	0.01	0.01	0.00	0.02	0.01	0.01	0.00	0.03	0.02	0.02	0.00	0.04	0.04	0.04	0.01	0.08
Honey	0.00	0.00	0.00	0.00	0.00	0.00	0.00	0.00	0.01	0.00	0.00	0.01	0.01	0.00	0.00	0.01
Total dressing	0.04	0.12	0.02	0.19	0.04	0.11	0.02	0.17	0.05	0.11	0.02	0.18	0.07	0.12	0.02	0.21
Chocolates																
Chocolate with milk	0.00	0.01	0.01	0.02	0.00	0.01	0.01	0.02	0.00	0.02	0.01	0.03	0.00	0.02	0.02	0.04
Black chocolate	0.01	0.03	0.02	0.05	0.01	0.03	0.02	0.06	0.01	0.03	0.02	0.07	0.02	0.04	0.02	0.08
Chocolate with almonds	0.00	0.01	0.00	0.01	0.00	0.01	0.01	0.02	0.01	0.02	0.01	0.04	0.01	0.03	0.02	0.05
White chocolate	0.00	0.00	0.00	0.00	0.00	0.00	0.00	0.00	0.00	0.00	0.00	0.00	0.00	0.01	0.00	0.01
Cocoa powder	0.02	0.04	0.02	0.08	0.02	0.06	0.03	0.11	0.02	0.06	0.03	0.11	0.02	0.05	0.03	0.09
Total Chocolates	0.03	0.09	0.05	0.17	0.04	0.11	0.06	0.21	0.04	0.13	0.08	0.25	0.04	0.15	0.08	0.27
Total Polyamine Intake	74.61	31.90	20.58	127.09	93.11	38.98	23.98	156.07	157.48	62.59	36.04	256.11	252.77	92.51	47.74	393.03

PU = putrescine; SPD = spermidine; SPM = spermine
